# UPLC-MS/MS method for simultaneously determining nucleosides and methyl-nucleosides in liver mRNA of Epimedin C-induced liver injury mouse model

**DOI:** 10.1186/s13020-021-00501-7

**Published:** 2021-09-21

**Authors:** Zhizhen Song, Zeyun Li, Xueqian Wen, Ruijuan Liu, Xin Tian

**Affiliations:** 1 Department of Pharmacy, The First Affiliated Hospital of Zhengzhou University, Zhengzhou, People’s Republic of China; 2 Henan Key Laboratory of Precision Clinical Pharmacy, Zhengzhou, People’s Republic of China

**Keywords:** UPLC-MS/MS, Epimedin C, Liver injury, Nucleosides, mRNA methylation

## Abstract

**Background:**

Epimedin C, one of the main active ingredients of Epimedium, has been reported to have potential hepatotoxicity. However, the mechanism of Epimedin C-induced liver injury has not been studied. mRNA methylation, mainly including *N*6-methyladenosine and *N*5-methylcytidine, is implicated in the regulation of many biological processes and diseases. The study of quantifying mRNA methylation alterations in Epimedin C-induced liver injury mice may contribute to clarify the mechanism of its hepatotoxicity. Therefore, an analysis method needs to be established to determine nucleoside and methyl-nucleoside levels in liver mRNA.

**Methods:**

An ultra-high performance liquid chromatography-tandem mass spectrometry (UPLC-MS/MS) method was developed and validated to simultaneously determine six nucleosides (adenosine, uridine, cytidine, guanosine, *N*6-methyladenosine and *N*5-methylcytidine) in liver mRNA. Besides, the Epimedin C-induced liver injury mouse model was studied by intragastrical administration Epimedin C at a daily dose of 10 or 40 mg/kg for 4 weeks. The nucleoside samples of the mice liver mRNA were prepared and separated on an UPLC column using 0.1% formic acid water and methanol after enzymatic digestion. Then the sample was detected by a Qtrap 6500 mass spectrometer.

**Results:**

In this method, calibration curves of the six nucleosides showed good linearity over their concentration ranges. The linear ranges were 40–20,000 pg/mL for adenosine, cytidine, *N*6-methyladenosine and *N*5-methylcytidine, 0.2–100 ng/mL for guanosine, and 2–1000 ng/mL for uridine. Epimedin C-induced liver injury mouse model was successfully established,which could be proved by the elevation of serum aminotransferase levels, and the increased inflammatory cell infiltration as well as vacuolar degeneration in liver. The *N*6-methyladenosine and *N*5-methylcytidine levels, and the ratios of *N*6-methyladenosine to adenosine and *N*5-methylcytidine to cytidine of the mice liver mRNA were all significantly increased after Epimedin C treatment.

**Conclusion:**

The established method was successfully applied to the determination of six nucleosides levels in liver mRNA of the Epimedin C-induced liver injury mice model and the control group. The results indicated that mRNA methylation might be associated with Epimedin C-induced liver injury. This study will facilitate the mechanism research on the hepatotoxicity of Epimedin C.

## Background

In recent years, the incidence of drug-induced liver injury has been dramatically increased due to the surging demand for herbal medicine and health care products [1]. Epimedium, an ancient herb medicine, was wildly used in Asia countries. In China, many herbal prescriptions containing Epimedium have been used for rheumatism, arthritis, osteoporosis, and other diseases [2, 3]. In the past few years, varying degrees of liver damage have been frequently reported in Epimedium consuming patients [4, 5]. However, few studies have been conducted on potential mechanism and underlying substance for its hepatotoxicity. As one of the main active ingredients and quality indicator of Epimedium, Epimedin C has shown strong cytotoxicity to HL-7702 and HepG2 cells [6–8]. Therefore, Epimedin C has been proposed as the substance responsible of Epimedium related liver injury. As far as we known, no Epimedin C-induced liver injury has been reported in animal models, which shall prompt investigation of underlying mechanisms.

Methylation modifications of messenger RNAs (mRNA), another layer of epigenetic regulation in addition to DNA and histone modifications, include *N*6-methyladenosine (m^6^A), *N*5-methylcytidine (m^5^C), *N*1-methyladenosine (m^1^A), *N*7-methylguanosine (m^7^G), pseudouridine (ψ), and so on [9, 10]. Among the methylation modifications of mRNA, m^6^A and m^5^C are the most common internal modifications in eukaryotic mRNA, and have been implicated in a variety of biological processes and diseases [9–11]. For example, m^6^A alteration was associated with the progression and metastasis of hepatocellular carcinoma [12, 13], and acetaminophen-induced liver injury in mice [14]. Besides, m^5^C played an important role in regulation of cell stress response, apoptosis, and hepatocellular carcinoma [15, 16]. However, the relationship between mRNA methylation and Epimedin C-induced liver injury is still unknown. It was expected that revealing of m^6^A and m^5^C alterations in Epimedin C-induced liver injury may contribute to clarify the liver toxicity mechanism of Epimedin C. In consequence, the content of m^6^A and m^5^C in liver mRNA of Epimedin C-induced liver injury mice needs to be quantified.

To the best of our knowledge, some liquid chromatography–tandem mass spectrometry (LC-MS/MS) methods have been reported for the determination of m^6^A or m^5^C in total RNA or mRNA [14, 17–22]. However, there are some limitations in those methods. For example, the run time of one sample was long [20], or it need a large amount of mRNA [21, 22], or the lower limit of quantification was high [14]. Therefore, a rapid and sensitive ultra-high performance liquid chromatography-tandem mass spectrometry (UPLC-MS/MS) method for determination of six nucleosides in mRNA is necessary to be developed for our study.

In this study, the Epimedin C-induced liver injury mouse model was established, and a selective, rapid and sensitive UPLC-MS/MS method was developed for simultaneous determination of six nucleosides in liver mRNA of the mouse model. This work may provide a new idea for further research on the mechanism of Epimedin C or herbal medicine-induced liver injury.

## Materials and methods

### Chemicals and reagents

Epimedin C (purity ≥ 98%), used as the dosage administration, was purchased from Cdmust Biology Technology Ltd. (Chengdu, China). Lamivudine (internal standard, IS, Batch No. J0820AS, purity > 99%) was purchased from Meilun Biotechnology company Ltd. (Dalian, China). Adenosine (Batch No. NFVEH-MG, purity > 99%), guanosine (Batch No. DLJPH-CK, purity > 98%), and cytidine (Batch No. 5PV0G-QR, purity > 98%) were purchased from Tokyo Chemical Industry (Shanghai) (Shanghai, China). Uridine (Batch No. LM90Q27, purity 99%), m^6^A (Batch No. LK70U69, purity 97%), and m^5^C (Batch No. L370O143, purity 98%) were purchased from J&K Chemical (Shanghai, China). Nuclease P1 and alkaline phosphatase were purchased from Takara Biotechnology (Dalian, China). Methanol (HPLC grade) and Formic acid (chromatographic grade) were obtained from Fisher Scientific (Shanghai, China). Ultra-purified water was used throughout this study and was prepared using a Milli-Q purification system (Millipore, Milford, MA, USA). All of the other chemicals and reagents were of analytical grade.

### Animals

Male Balb/c mice (6–8 weeks old and weighing 18.0–22.0 g) were obtained from Beijing HFK bioscience Co., Ltd. (Beijing, China). Mice were kept in cages under controlled conditions of 22 ± 0.5 °C, 50 ± 2.0% RH and maintained with free access to standard laboratory food and water for 1 week before experiments.

### Establishment of the Epimedin C-induced liver injury model and experimental groups

Animals were randomly divided into three groups (n = 7 each): the normal control group, the Epimedin C (10 mg/kg) group, and the Epimedin C (40 mg/kg) group. Epimedin C were completely dissolved in 0.9% saline before passing through a 0.22 μm cell strainer. The Epimedin C was intragastrically administered at a single dose of 10 or 40 mg/kg body weight per day. Meanwhile, the normal control mice were given the same volume of saline. After 4 weeks of intragastric administration, all mice were sacrificed, then blood samples and liver tissues were collected. The procedures for the present study were approved by the Guide for the Care set by the National Institutes of Health.

### Assessment of liver injury

Serum alanine transaminase (ALT) and aspartate transaminase (AST) levels in serum were analyzed using colorimetric tests (Nanjing Jiancheng Bioengineering Research Institute, Nanjing, China). Liver tissues fixed in 4% paraformaldehyde were embedded in paraffin using a tissue procedure, and 4-μm-thick slices were prepared and stained with hematoxylin and eosin (H&E) reagent. Photomicrographs were observed with a light microscope to evaluate liver injury.

### UPLC-MS/MS instruments and conditions

The LC was performed using an ExionLC™ analytical (UPLC) system (AB Sciex, USA). Chromatographic separation was carried out on a Kinetex® 2.6 μm Polar C18 100A LC column (100 mm × 2.1 mm i.d.). The flow rate was 0.3 mL/min. The mobile phase included ultra-purified water containing 0.1% formic acid (solvent A) and methanol (solvent B) in a linear gradient. The gradient program was as follows: 0 to 0.5 min, 95% A; 0.5 to 3 min, 95 to 30% A; 3 to 4 min, 30% A; 4 to 4.1 min, 30 to 95% A; 4.1 to 6 min, 95% A. The injection volume was 10 μL and the total run time was 8 min. The temperature of the autosampler was set at 4 °C, and the column temperature was maintained at 40 °C. MS/MS analysis was carried out on a Qtrap 6500 mass spectrometer (AB Sciex, Redwood City, CA, USA) equipped with Turbo Ionspray interface operating in positive ESI mode. The instrument was operated with an ion spray voltage of 4.5 kV and a heater gas temperature of 500 °C. Mass-dependent parameters (declustering potential, entrance potential, collision energy, and collision cell exit potential) were set to the optimal values obtained by automated optimization. Data acquisition was achieved by multiple reaction monitoring (MRM). The precursor-product ion pair and the optimal values of mass parameters are listed in Table 1. Positive ion mode was used and the dwell time was set at 100 ms. Data acquisition was generated and processed using the Analyst 1.6.2 software (AB Sciex).

**Table 1:** Multiple reaction monitoring transitions and optimized mass parameters for the analytes

Analytes	Precursor ion (m/z)	Product ion (m/z)	DP (V)	EP (V)	CE (V)	CXP (V)
Adenosine (A)	268.1	136.1	30	10	22	10
Uridine (U)	245.0	113.1	12	10	12	10
Cytidine (C)	244.1	112.0	30	10	13	10
Guanosine (G)	284.2	152.1	30	10	15	10
*N*6-Methyladenosine (m^6^A)	282.2	150.2	30	10	24	10
*N*5-Methylcytidine (m^5^C)	258.2	126.1	30	10	15	10
Lamivudine (IS)	230.2	112.0	30	10	14	10

*DP* declustering potential, *EP* entrance potential, *CE* collision energy, *CXP* collision cell exit potential

### Preparation of calibration standards and QC samples

Stock solutions for calibration and quality control (QC) were accurately weighed and dissolved in dimethylsulfoxide (2% of the total volume) before adding an appropriate volume of methanol to final concentration of 1 mg/mL. Working solutions were prepared by serially diluting the stock solutions with water, and then the corresponding working solutions were mixed to prepare mixed working solutions with concentration in the ranges of 160–80,000 pg/mL for A, C, m^5^C and m^6^A, 0.8–400 ng/mL for G, and 8–4000 ng/mL for U. The stock solution (1 mg/mL) of the IS was dissolved in water to 4 ng/mL containing 0.4% formic acid. All solutions were kept at − 20 °C and brought to room temperature before use. The calibration standards were prepared by spiking 7.5 μL of the corresponding working solutions mentioned above into 22.5 μL of mixtures of nuclease P1 (0.1 U) and alkaline phosphatase (2 U) to yield concentrations of 40, 120, 500, 1000, 2000, 4000, 8000 and 20,000 pg/mL for A, C, m^5^C and m^6^A, 0.2, 0.6, 2.5, 5, 10, 20, 40 and 100 ng/mL for G, and 2, 6, 25, 50, 100, 200 400 and 1000 ng/mL for U. The QC samples were prepared in the same way as the calibration samples at three concentrations 120, 1600, 16,000 pg/mL for A, C, m^5^C and m^6^A, 0.6, 8, 80 ng/mL for G, 6, 80, 800 ng/mL for U.

### RNA isolation from liver tissues and Enzymatic digestion of the mRNA

100 mg of liver tissue was completely disrupted and homogenized into 1 mL TRIzol reagent. Then, the total RNA of liver tissue was isolated according to the manufacturer’s instructions. After analyzed by a NanoDrop One (Thermo Scientific), the Dynabeads® mRNA Purification Kit (Ambion) was used to enrich mRNA. The contaminant DNA was removed from the mRNA samples by using DNase, and the concentration was analyzed by a Qubit 3.0 Fluorometer (Invitrogen).

Referring to the methods reported, enzymatic digestion of the mRNA was performed [14]. The mixture sample was made included 100 ng mRNA, 1 μL of nuclease P1 (0.1 U/μL, Takara), and 2 μL of alkaline phosphatase (calf intestine, 1 U/μL, Takara), and the total volume was brought to 30 µL with ultrapure distilled water. After fully vortexed, the mixtures were incubated at 37 °C for 12 h. Then, 10 μL IS solution (IS, lamivudine, 4 ng/mL) containing 0.4% formic acid was added. The mixtures were vortexed for 15 s and transferred into ultrafiltration tubes (MW cutoff of 3 kDa, Pall Corporation), and centrifuged at 4 °C, 14,000×*g* for 15 min. The filtrate was added to an autosampler vial, then 10 μL of the filtrate was used for UPLC-MS/MS analysis.

### Method validation

The analytical methodology was under the guidelines set by the United States Food and Drug Administration [23] and the Chinese Pharmacopoeia Commission [24].

The selectivity was evaluated by comparing chromatograms of mRNA-free blank enzymolysis matrix, blank enzymolysis matrix with all analytes, and a liver mRNA enzymolysis sample containing IS from a mouse after Epimedin C treatment. The absence of peaks at retention times of seven analytes indicated no interference in the test samples.

After detection of the upper limit of the quantification (ULOQ) samples, blank samples were injected to evaluate the carryover effects. The response peak of any analytes must be < 10% of the lower limit of the quantification (LLOQ) samples.

The linearity was investigated by plotting the peak-area ratios of the analytes (A, U, C, G, m^6^A and m^5^C) to the IS versus the concentrations of the calibration standards. The calibration equations were fitted using a weighed least-squares linear regression analysis (weighing factor of 1/x^2^). The accuracy, expressed as the mean relative error (RE, %), should be ≤ 20% for LLOQ and ≤ 15% for the other seven concentrations of the calibration standards.

To assess the precision and accuracy of the method, five replicates of QC (at three concentration levels) and LLOQ were prepared and analyzed within 3 validation days. Both the accuracy (RE, %) and intra- and inter-precision (RSD, %) for LLOQ should be less than or equal to 20%. The accuracy and precision for the QC levels should be within ± 15%.

To determine whether matrix components affected the ion suppression or enhancement in the method, the matrix effect (ME) was assessed by comparing the corresponding peak area responses of enzymolysis matrix with all analytes and the blank samples in which the enzymolysis matrix was replaced with water. In this method, the variability values of the MEs (RSD, %) should be less than 15%.

To evaluate the stability of the analytes in the enzymolysis matrix during sample preparation and storage, the low and high QC concentration levels in different storage conditions were detected. The storage conditions included room temperature for 6 h, three freeze–thaw cycles, autosampler at ambient temperature for 20 h, and freezing at − 20 °C for 30 days. The analytes were stable when 85–115% of the initial concentration was retained.

The dilution integrity was assessed by testing the solution which was diluted 100-fold with blank enzymolysis matrix before ultra-filtration from highly concentrated samples above the upper limit of standard curves. The accuracy and precision should be within ± 15%.

### Data analysis

The data were presented as the arithmetic mean ± SD. Statistical analyses were performed using SPSS software for Windows. Statistical significance was assessed by unpaired two-tailed Student’s t-test between two samples. *p* value < 0.05 was considered statistically significant.

## Results

### Chromatography and mass spectrometry

**Fig. 1:**
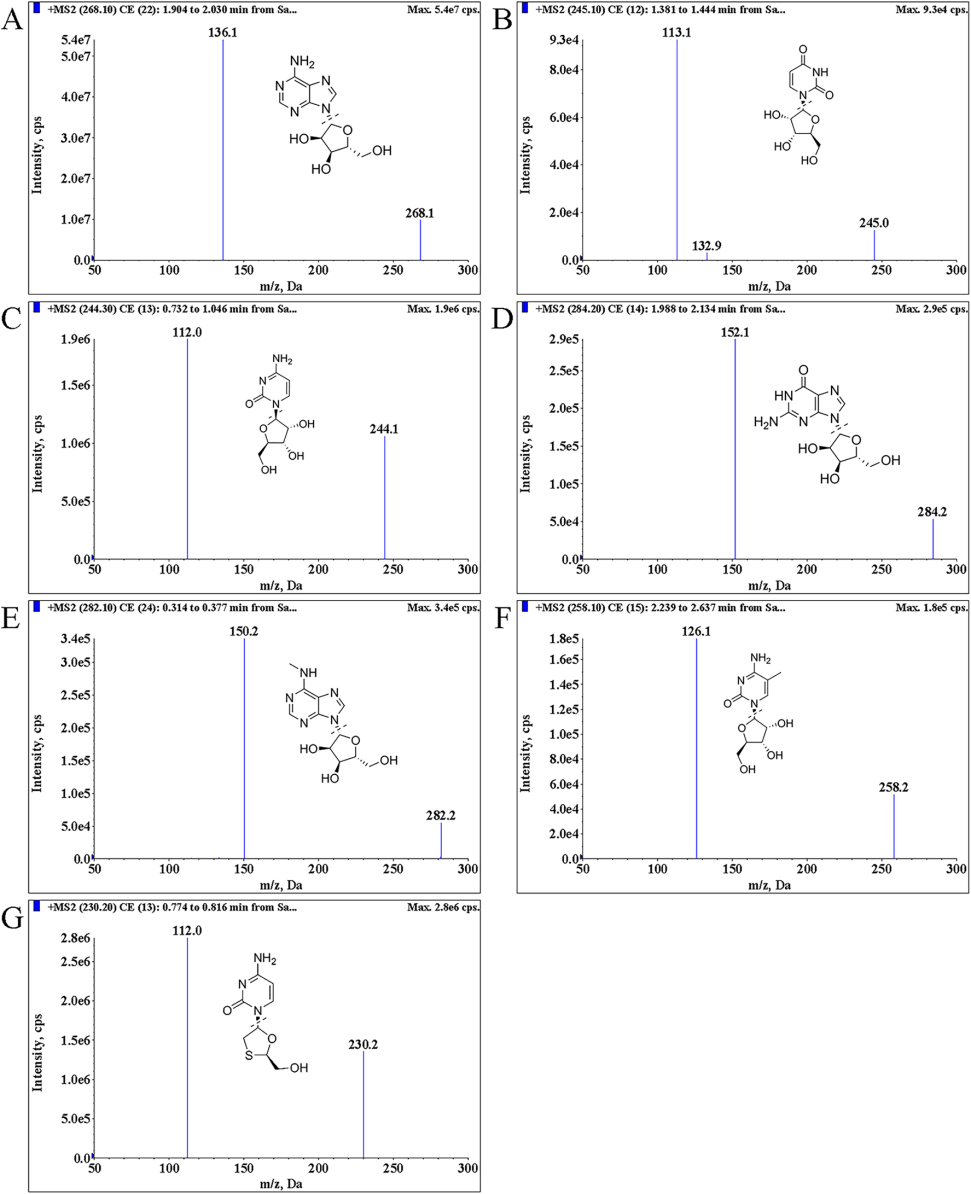
Chemical structures and product ion spectra. **A** A; **B** U; **C** C; **D** G; **E** m^6^A; **F** m^5^C; **G** lamivudine

The chromatographic conditions of an UPLC-MS/MS method were important for the chromatographic behavior of the analytes. A Kinetex® 2.6 μm Polar C18 100A LC column was proved to be more suitable for the separation of the targeted compounds in the sample. The composition of mobile phase and the gradient elution programs were optimized to obtain better sensitivity, better peak shape, and mass response to the analytes. Ionization was achieved using the positive ion-monitoring mode with ESI. The precursor ions and product ions of six nucleosides and lamivudine (IS) for the MRM transition were obtained. The MS/MS product ion spectra of the analytes are shown in Fig. 1.

### Method validation

#### Selectivity, linearity, and carryover

**Fig. 2:**
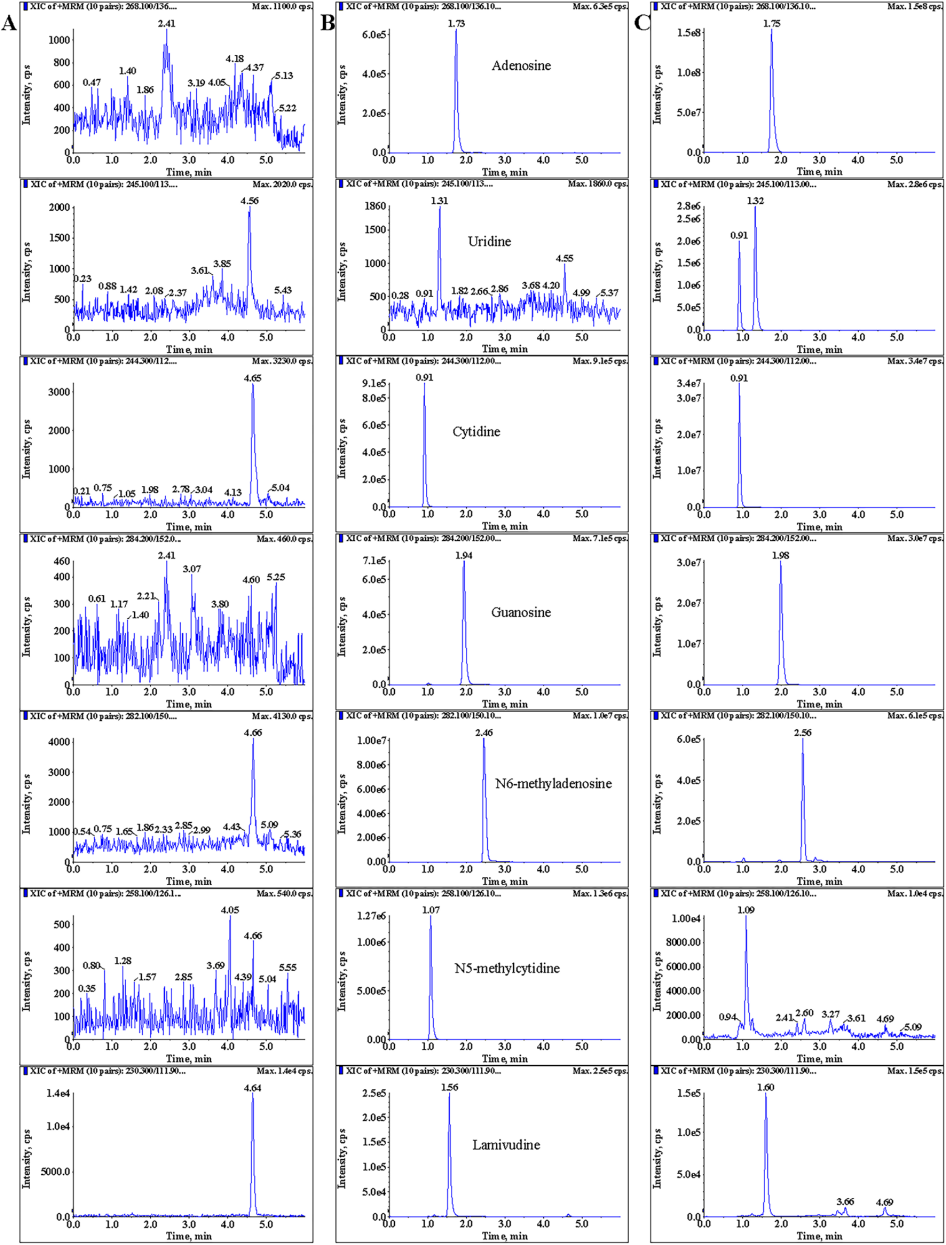
Typical MRM chromatograms of A, U, C, G, m^6^A, m^5^C and lamivudine (IS). **A** Blank enzymolysis matrix sample; **B** blank enzymolysis sample mixed with the six nucleosides and IS; **C** a liver mRNA enzymolysis matrix sample from Epimedin C-treated mouse

Typical chromatograms of six nucleosides and lamivudine (IS) are shown in Fig. 2. No significant interference was observed at the retention times of A, U, C, G, m^6^A, m^5^C and IS which were 1.73, 1.31, 0.91, 1.94, 2.46, 1.07 and 1.56 min, respectively. The calibration curves were linear over the concentration ranges of 40–20,000 pg/mL (*r* > 0.99, n = 8) for A, C, m^5^C and m^6^A, 0.2–100 ng/mL for G, and 2–1000 ng/mL for U (*r* > 0.99, n = 8). Carryover effects were absent for analytes.

#### Accuracy and precision

Three batches of LLOQ and QC samples were evaluated to obtain the intra- and interday precision and accuracy with the current method. The validation results of accuracy and precision for A, U, C, G, m^6^A and m^5^C are listed in Table 2, which demonstrated that the values of RSD and RE were all inside the acceptable variability limits. It indicates that the method is accurate and precise.

**Table 2:** Precision and accuracy data for the quantification of six nucleosides in mRNA (n = 5)

Analytes	Concentration levels (mean ± SD, pg/mL)		RSD (%)		RE (%)
	Added	Measured	Intra-day	Inter-day	Accuracy
Adenosine (A)	40	40.8 ± 1.9	6.5	4.3	2.0
	120	133.3 ± 3.1	3.9	2.0	11.1
	1600	1792.7 ± 33.7	3.6	1.4	12.0
	16,000	15,120.0 ± 270.4	4.0	1.0	− 5.5
Uridine (U)	2000	1919.3 ± 96.0	6.5	4.7	− 4.0
	6000	6101.3 ± 220.7	7.4	2.5	1.7
	80,000	83,680.0 ± 1235.9	2.6	1.2	4.6
	800,000	710,133.3 ± 10,405.1	2.1	1.3	− 11.2
Cytidine (C)	40	36.7 ± 2.3	0.5	6.8	− 8.3
	120	117.3 ± 5.4	4.8	4.5	− 2.3
	1600	1672.7 ± 26.0	1.5	1.6	4.5
	16,000	15,953.3 ± 247.5	1.2	1.6	− 0.3
Guanosine (G)	200	195.0 ± 12.0	12.7	4.1	− 2.5
	600	630.4 ± 21.8	6.4	2.7	5.1
	8000	8472.0 ± 280.3	4.3	3.1	5.9
	80,000	78,180.0 ± 1894.1	4.5	1.9	− 2.3
*N*6-Methyladenosine (m^6^A)	40	36.2 ± 2.0	13.8	1.9	− 9.5
	120	131.2 ± 6.9	2.4	5.6	9.3
	1600	1733.3 ± 96.0	7.2	5.2	8.3
	16,000	15,806.1 ± 196.9	1.2	1.2	− 1.2
*N*5-Methylcytidine (m^5^C)	40	38.4 ± 1.6	2.1	4.3	− 4.0
	120	122.3 ± 3.3	4.2	2.3	1.9
	1600	1697.3 ± 39.9	6.0	0.6	6.1
	16,000	15,726.7 ± 286.5	3.3	1.4	− 1.7

#### Matrix effects and stability

The results of ME and extraction recovery were evaluated using QC samples at the low and high QC levels. The IS-corrected matrix factors of the six different batches of enzymolysis matrix at low- and high-concentrations were 96.1 ± 3.1 and 97.8 ± 3.1% for A, 105.4 ± 8.3 and 100.0 ± 3.2% for U, 100.2 ± 7.5 and 97.1 ± 4.2% for C, 100.1 ± 4.6 and 98.6 ± 4.9% for G, 98.3 ± 3.0 and 98.5 ± 3.7% for m^6^A, and 100.5 ± 5.0 and 95.0 ± 3.9% for m^5^C, respectively. These data indicated that the MEs for all the analytes were negligible following the current method.

The stability results of the analytes are summarized in Table 3. A, U, C, G, m^6^A and m^5^C remained stable at room temperature for 6 h and after three freeze–thaw cycles. All the analytes were also stable in the autosampler at ambient temperature for 20 h and after freezing at − 20 °C for 30 days.

**Table 3:** The stability test results in enzymolysis matrix under various storage conditions (n = 5)

Analytes	Added (pg/mL)	Room temperature for 6 h		Autosampler for 20 h (RT)		Three freeze–thaw cycles		Freezing for 30 days (− 20℃)	
		Measured (pg/mL)	RE (%)	Measured (pg/mL)	RE (%)	Measured (pg/mL)	RE (%)	Measured (pg/mL)	RE (%)
Adenosine	120	130.0 ± 2.1	8.3	133.0 ± 1.0	10.8	134.2 ± 3.6	11.8	133.0 ± 1.6	10.8
	16,000	15,360.0 ± 230.2	− 4.0	15,380.0 ± 164.3	− 3.9	15,240.0 ± 54.8	− 4.8	14,840.0 ± 167.3	− 7.3
Uridine	6000	6316.0 ± 170.7	5.3	6052.0 ± 169.9	0.9	5986.0 ± 141.2	− 0.2	5868.0 ± 140.2	− 2.2
	800,000	706,000.0 ± 12,308.5	− 11.8	688,800.0 ± 6760.2	− 13.9	687,200.0 ± 15,073.2	− 14.1	716,600.0 ± 13,794.9	− 10.4
Cytidine	120	120.8 ± 2.9	0.7	121.0 ± 1.9	0.8	119.0 ± 4.4	− 0.8	117.2 ± 4.1	− 2.3
	16,000	16,140.0 ± 240.8	0.9	16,040.0 ± 230.2	0.3	16,080.0 ± 238.7	0.5	15,980.0 ± 228.0	− 0.1
Guanosine	600	653.0 ± 16.3	8.8	625.8 ± 14.8	4.3	628.2 ± 14.8	4.7	605.2 ± 28.1	0.9
	80,000	79,200.0 ± 748.3	− 1.0	77,520.0 ± 1158.4	− 3.1	76,300.0 ± 1647.7	− 4.6	77,320.0 ± 2008.0	− 3.4
m^6^A	120	132.4 ± 3.0	10.3	126.0 ± 3.4	5.0	123.8 ± 3.0	3.2	132.2 ± 12.1	10.2
	16,000	15,962.0 ± 205.7	− 0.2	15,561.0 ± 218.9	− 2.7	15,490.8 ± 269.4	− 3.2	15,841.8 ± 281.8	− 1.0
m^5^C	120	123.4 ± 3.8	2.8	121.6 ± 2.2	1.3	120.0 ± 2.2	0	122.2 ± 3.7	1.8
	16,000	15,720.0 ± 130.4	− 1.8	15,600.0 ± 100.0	− 2.5	15,600.0 ± 308.2	− 2.5	15,980.0 ± 249.0	− 0.1

#### Dilution integrity

Results of the dilution integrity were shown in Table 4. The precision and accuracy of the dilution test at the low and high concentration levels were within the acceptable criteria, indicated that A, U, C, G, m^6^A and m^5^C were assayed reliably by diluting 100-fold with blank enzymolysis matrix. Samples could be tested by dilution when the analyte concentration exceeded the linear range of the standard curve.

**Table 4:** Precision and accuracy of the dilution QC samples (n = 5)

Analytes	Added (ng/mL)	Dilution factor	Caculated (pg/mL)	Measured (pg/mL)	RSD (%)	RE (%)
Adenosine	12	100	120	137.8 ± 2.2	1.6	14.8
	1600	100	16,000	14,940.0 ± 167.3	1.1	− 6.6
Uridine	600	100	6000	5862.0 ± 99.3	1.7	− 2.3
	80,000	100	800,000	716,000.0 ± 10,583.0	1.5	− 10.5
Cytidine	12	100	120	115.8 ± 5.4	4.6	− 3.5
	1600	100	16,000	16,100.0 ± 339.1	2.1	0.6
Guanosine	60	100	600	635.2 ± 26.0	4.1	5.9
	8000	100	80,000	78,920.0 ± 1856.6	2.4	− 1.4
m^6^A	12	100	120	126.4 ± 10.2	8.0	5.3
	1600	100	16,000	15,631.2 ± 212.5	1.4	− 2.3
m^5^C	12	100	120	125.8 ± 4.5	3.6	4.8
	1600	100	16,000	16,540.0 ± 320.9	1.9	3.4

### Epimedin C-induced liver injury

**Fig. 3:**
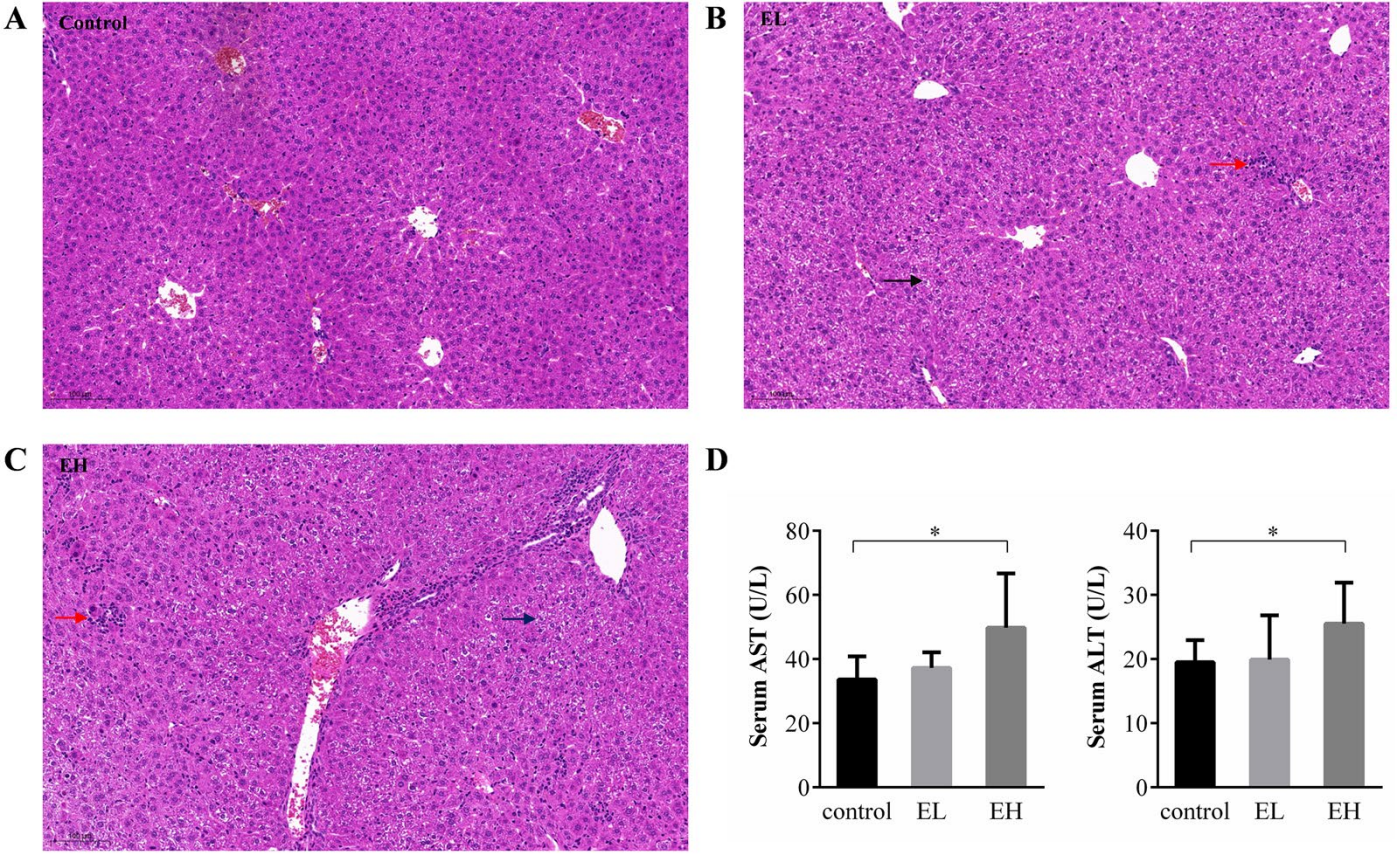
Representative photomicrographs of liver histopathological examination and serum aminotransferase levels after Epimedin C treatment. **A** Epimedin C was absent in the normal control group; **B** the mice were treated with Epimedin C (10 mg/kg) in the EL group; **C** the mice were treated with Epimedin C (40 mg/kg) in the EH group. *P < 0.05 vs. the normal control group. The black arrow represents the vacuolar degeneration and the red arrow stands for the inflammatory cell infiltration

Hematoxylin and eosin staining of the liver was performed in order to evaluate the pathological changes in liver. Epimedin C increased the inflammatory cell infiltration (the red arrow) and vacuolar degeneration (the black arrow) observed in liver, and these effects were induced with the increased dose of Epimedin C (Fig. 3). Serum aminotransferase levels were in good agreement with histopathological changes in our study. The serum levels of transaminase were measured to evaluate hepatocellular damage. The results showed that serum ALT and AST levels were significantly increased after oral administration of 40 mg/kg Epimedin C compared with the normal control group (Fig. 3).

### Application for quantifying the nucleosides

**Fig. 4:**
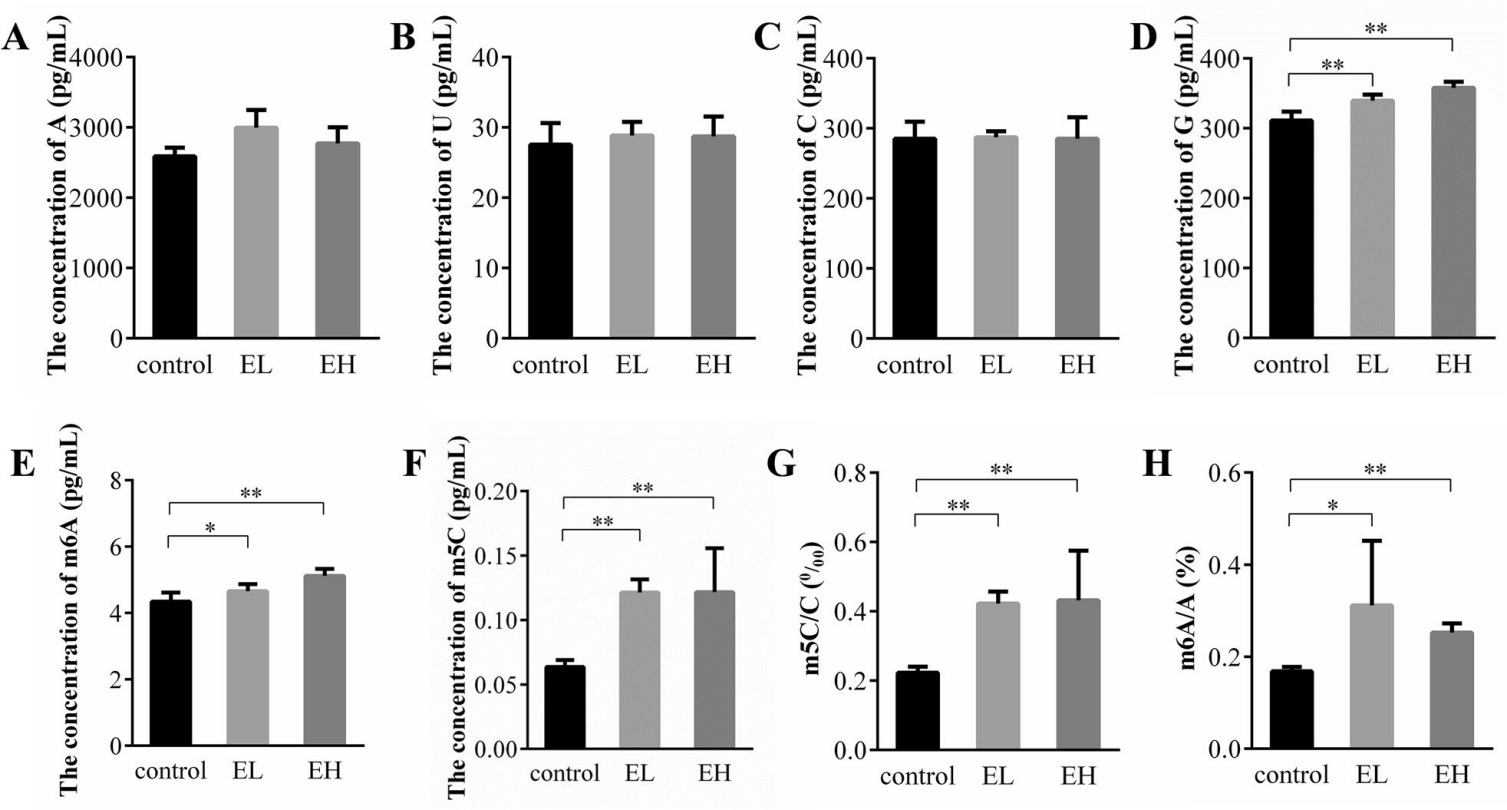
Concentration levels of six nucleosides and relative contents of m^6^A and m^5^C in test samples. The concentration levels of **A** A, **B** U, **C** C, **D** G, **E** m^6^A and **F** m^5^C in test samples; **G**, **H** the content of modified nucleoside m^6^A (ratio of m^6^A/A) and m^5^C (ratio of m^5^C/C). Epimedin C was absent in the normal control group. The mice were treated with Epimedin C (10 mg/kg) in the EL group. The mice were treated with Epimedin C (40 mg/kg) in the EH group. *P < 0.05 and **P < 0.01 vs. the normal control group

The mean concentration levels of m^6^A and m^5^C in prepared test samples of mice liver mRNA in the control and Epimedin C-induced liver injury model groups are presented in Fig. 4. The ratios of m^6^A to A (m^6^A/A% = C_m_6_A_/C_A_ × %) and m^5^C to C (m^5^C/C% = C_m_5_C_/C_C_ × %) expressed the content of modified nucleoside in mouse liver mRNA. The results showed that both the concentration levels of m^6^A and m^5^C, and the ratio of m^6^A/A and m^5^C/C in the mRNA of in Epimedin C-induced mice live injury mice were significantly increased when compared with the normal control mice. According to these results, it could be indicated that epigenetic modification in mice liver were impacted by Epimedin C treatment.

## Discussion

Some studies indicated that the Epimedin C had the potential hepatotoxicity [6–8], but the mechanism of Epimedin C-induced liver injury has not been studied. In this study, the Epimedin C-induced liver injury mouse model was established for the first time. The oral dose (10 mg/kg, equivalent dose) of Epimedin C in mouse model is calculated according to the clinical dosage of Epimedin C in the Xian-Ling-Gu-Bao capsule. Meanwhile, four times equivalent dose (40 mg/kg) of Epimedin C was also investigated for the liver injury mouse model. The results showed that the mouse model was induced successfully by intragastrical administration Epimedin C at a daily dose of 40 mg/kg for 4 weeks in mice. The mouse model provides useful tools for evaluating the hepatotoxicity and mechanism of Epimedin C in future studies.

To the best of our knowledge, methylation modification disorders of mRNA are closely related to the occurrence and development of many liver diseases. The study of investigating the connection of mRNA methylation and Epimedin C-induced liver injury was performed. A new UPLC-MS/MS method was developed for simultaneously determination of six nucleosides in mice liver mRNA. Compared to previous reports, this present method has higher sensitivity, wider linear range, and more analytes. Due to consisting of multiple compounds in mRNA, the simultaneous quantitation of six nucleoside encounters the great challenge in short running time using UPLC. Multiple reaction monitoring, a highly specific technique, was used for quantifying the targeted analyte without the considering of baseline chromatographic separation [25]. Furthermore, ultra-purified water containing 0.1% formic acid/methanol in a linear gradient was the optimum mobile phase to achieve the chromatogram. Because of the much higher concentrations of A, U, C and G than that of m^6^A and m^5^C, different ranges were designed for the standard curves. In addition, saturation nonlinearity is unavoidable in the highly sensitive mass spectrometric detector, when the concentrations of analytes are too high. In this study, the concentrations of A, U, C and G in the samples were above the upper limit of standard curves. Therefore, the samples were diluted 100-fold with blank enzyme matrix when detecting A, U, C and G.

Although one study has reported the content of m^6^A in liver mRNA of drug-induced liver injury mouse model [14], m^5^C alteration in liver injury mouse model is discovered firstly in our study. Additionally, it is the first time to reveal mRNA methylation alteration in liver mRNA of liver injury mice induced by Chinese herbal medicine. Our study validates the changes of m^6^A and m^5^C in mice liver mRNA after Epimedin C treatment, but the relationship between modified nucleosides and the mechanism of Epimedin C-induced liver injury is not well understood, which requires further investigation. This study may offer a new idea and approach for studying the mechanism of Epimedin C-induced liver injury.

## Conclusions

An UPLC-MS/MS method for the simultaneous determination of A, U, C, G, m^6^A and m^5^C in mRNA was presented and validated. The method was applied to the detection of six nucleosides in liver mRNA of Epimedin C-induced liver injury mice. The results demonstrated that epigenetic modification changed in mice liver after Epimedin C treatment with test dose, and the m^6^A and m^5^C might be associated with Epimedin C-induced liver injury, which were reported for the first time. Our study provides a new idea for further research on the mechanism of Epimedin C or herbal medicine-induced liver injury.

## Data Availability

The datasets supporting the conclusion of this article are included within the article and its additional files.

## Abbreviations

A: Adenosine
U: Uridine
C: Cytidine
G: Guanosine
m^6^A: *N*6-Methyladenosine
m^5^C: *N*5-Methylcytidine
ME: Matrix effect
MRM: Multiple reaction monitoring
mRNA: Messenger ribonucleic acid
ALT: Alanine transaminase
AST: Aspartate transaminase
UPLC-MS/MS: Ultra-high performance liquid chromatography tandem mass spectrometry
QC: Quality control
